# Draft genome sequence of *Pelosinus* sp. IPA-1, a bacterium isolated from arsenic-contaminated soil in Japan

**DOI:** 10.1128/MRA.00323-23

**Published:** 2023-07-24

**Authors:** Masashi Kuroda, Shigeki Yamamura, Nobuyoshi Nakajima, Seigo Amachi

**Affiliations:** 1 Faculty of Social and Environmental Studies, Tokoha University, Shizuoka, Japan; 2 Regional Environment Conservation Division, National Institute for Environmental Studies, Tsukuba, Japan; 3 Biodiversity Division, National Institute for Environmental Studies, Tsukuba, Japan; 4 Graduate School of Horticulture, Chiba University, Matsudo, Japan; University of Southern California, Los Angeles, California, USA

**Keywords:** arsenic, draft genome

## Abstract

*Pelosinus* sp. strain IPA-1 is a bacterium isolated from arsenic-contaminated soil in Japan. We here report the draft genome sequence of strain IPA-1.

## ANNOUNCEMENT

Arsenic contamination of groundwater is a serious issue worldwide (1). Because soil microbial activities significantly affect the release of naturally occurring arsenic in the pedosphere into the hydrosphere (2), the microbial community in arsenic-contaminated soil is of great interest. *Pelosinus* sp. IPA-1 was isolated from an arsenic-contaminated soil (containing 1,801 mg/kg of As) of a former mine in Shimane, Japan, according to the method previously reported (3). Although the genus *Pelosinus* often predominates in enrichment cultures under high concentrations of metals/metalloids (3 - 5) and the chromate reduction by the strains of *Pelosinus* has been reported (5, 6), the role of these bacteria in an arsenic-contaminated environment is still unclear (3). Genomic data of strain IPA-1 will be a clue to revealing it.

Strain IPA-1 was cultivated for 3 days at 30°C in a minimal medium (7) containing 10 mM lactate as the sole electron donor and carbon source. A strict anaerobic technique was used in the preparation of the minimal medium. The cells were collected by centrifugation (7,200 × *g*, 4°C, 15 min). The genomic DNA of strain IPA-1 was extracted from the cells by using DNeasy Blood & Tissue Kit (Qiagen, Hilden, Germany). A paired-end DNA library (insert size, 330 bp) was constructed using the TruSeq DNA PCR-Free LT Library Prep Kit according to the supplier’s manual. Sequencing of the library with an Illumina Miseq System (Illumina, CA, USA) generated 3,021,484 reads with an average length of 248.74 bp. Low-quality sequences (<Q13) and adapters were trimmed using Trim Reads Tool of CLC Genomics Workbench v. 22.0 (CLC-GWB; Qiagen). The resulting 3,020,209 reads (750,043,179 bp in total) were assembled using the *De Novo* Assembly Tool of CLC-GWB with the following parameters: automatic bubble size: yes, minimum contig length: 500, word size: 64, perform scaffolding: yes, auto-detect paired distances: yes, mismatch cost: 2, insertion cost: 3, deletion cost: 3, length fraction: 0.5, and similarity fraction: 0.8. The draft genome sequence consisted of 36 contigs, a GC content of 38.5%, totaling 5,322,020 bp (corresponding to 141 × genome coverage of the trimmed reads), an N_50_ statistic of 597,585 bp, and a maximum contig size of 798,162 bp. A total of 5,082 coding sequences, 9 ribosomal RNAs, and 88 transfer RNAs were predicted by DDBJ Fast Annotation and Submission Tool (DFAST) v. 1.2.18 (8). The BLASTN analysis of the full-length 16SrRNA gene revealed that the closest relative of strain IPA-1 is *Pelosinus fermentans* DSM 17108^T^ with a nucleotide sequence identity of 98.5%, suggesting that strain IPA-1 is a member of the genus *Pelosinus*. Default parameters were used for all software unless otherwise specified.

## Data Availability

The draft genome sequence of *Pelosinus* sp. IPA-1 has been deposited in NCBI/ENA/DDBJ under accession numbers BSVC01000001 to BSVC01000036. Raw reads are available under accession number DRA015922. Bioproject and Biosample accession numbers are PRJDB14839 and SAMD00582791, respectively.
